# *TLR4* Receptor D299G/T399I Haplotype Polymorphism Is Associated with Insulin Resistance in Obese Female Subjects

**DOI:** 10.3390/genes11070814

**Published:** 2020-07-17

**Authors:** Elham Sharif, Mariam Al-Wakeel, Afnan Mohamed, Abdelhamid kerkadi, Nasser Rizk

**Affiliations:** 1 Biomedical Sciences Department, College of Health Sciences, QU Health, Qatar University, Doha 2713, Qatar; e.sharif@qu.edu.qa (E.S.); mariamalwakeel94@gmail.com (M.A.-W.); Afnan.biom@gmail.com (A.M.); 2 Human Nutrition Department, College of Health Sciences, QU Health, Qatar University, Doha 2713, Qatar; abdel.hamid@qu.edu.qa; 3 Biomedical Research Center, Qatar University, Doha 2713, Qatar; 4 Physiology Department, Mansoura Faculty of Medicine, Mansoura 35516, Egypt

**Keywords:** *TLR4* polymorphism, haplotype, insulin resistance and obesity

## Abstract

Background: Activation of Toll-like-receptor 4 (*TLR4*) causes chronic inflammation that can result in obesity and metabolic syndrome (MeS). Aim: This study aimed to investigate the role of *TLR4* polymorphisms of *TLR4*D299G/T399I, and its impact on protein expression of *TLR4* in obese female subjects. Methodology: A prospective cross-sectional association study was performed on Arab female subjects from Qatar University. The subjects were categorized according to BMI classifications into two groups: “obese; *n* = 69” and “non-obese; *n* = 136”. Anthropometric measurements, weight (kg), height (m) and waist circumference (WC) were evaluated, and the body mass index (BMI) was calculated. Fasting blood samples were collected, and assessment of glucose, lipid profile, C-reactive protein (CRP), leptin, IL-6 and insulin was performed. Insulin resistance was computed using HOMA-IR. Genotyping of the *TLR4* polymorphisms of *TLR4*D299G (rs4986790) and *TLR4*T399I (rs4986791) was performed by the 5′ nuclease assay by TaqMan MGB probe. Flow cytometry was used to evaluate the monocyte cell surface expression of *TLR4*. Results: The frequency distribution of the genotype revealed that homozygous AA is the most frequent among obese subjects (86.4%) for (*TLR4*D299G, A > G) and the homozygous CC genotype is the most frequent (92.4%) for (*TLR4*T399I, C > T). Haplotype analysis of *TLR4* D299G/T399I showed that GT carriers had a significant association with increased probability of insulin resistance (odds ratio = 4.73; 95% CI 1.19–18.90; *p*-value = 0.016). The monocyte cell surface of *TLR4* was significantly higher by 1.3 folds in obese compared to non-obese subjects. Conclusions: *TLR4* D299G/T399I haplotype polymorphism is associated with an increased risk of insulin resistance with the upregulation of *TLR4* protein expression in obese subjects.

## 1. Introduction

Obesity is a significant health concern to be addressed with a prevalence rate dramatically increasing worldwide over the last decades. Moreover, the World Health Organization (WHO) 2016 quantified that over 650 million adults 18 years or older were obese (13.0%), and 1.9 billion (39.0%) were overweight [1]. Obesity is linked with comorbidities such as hypertension, type 2 diabetes, cardiovascular disease (CVD), cancer and endocrine diseases [2]. In addition, it is linked with chronic low-grade inflammation that is known as meta-inflammation [3]. A cross-talk has been identified between adipose tissue and the innate immune system, which could lead to the development of obesity [4]. Metabolic syndrome (MeS) is a cluster of vascular and metabolic changes, including hypertension, fasting hyperglycemia, hypertriglyceridemia, low level of high-density lipoprotein cholesterol (HDL) and increased abdominal obesity (AO) [5]. Obesity and insulin resistance (IR) are critical factors which contribute to MeS development [6]. MeS increases the risk for type 2 diabetes mellitus and CVD development [7].

The toll like receptor (TLR) family includes pathogen-specific receptors expressed in numerous cell types. Toll like receptor 4 (TLR4) is a protein encoded with the gene of *TLR4*, which is located on chromosome 9 at the location 9q33.1 [8]. The TLR4 receptor is a pattern recognition receptor that can be triggered by lipopolysaccharide (LPS) [9] and saturated fatty acids (SFAs) [10] to participate in the secretion of critical pro-inflammatory cytokines that are important in the activation of a robust innate immune response [11]. Adipocytes express *TLR4*; once these *TLR4* are activated, it causes a pro-inflammatory state in adipose tissue via release of pro-inflammatory cytokine, thus initiating a potent immune response, which in turn may be involved in the developments of obesity and cardiometabolic syndrome [12]. Furthermore, experimental studies have revealed that disruption of the *TLR4* gene could protect against the inflammation and insulin resistance caused by obesity [13].

Among the various polymorphisms of *TLR4*, two SNPs (rs4986790 and rs4986791) produce single amino acid substitutes in exon 3 of the *TLR4* gene (*TLR4*) (rs4986790: D299G; rs4986791: T399I) in the extracellular *TLR4* domain [14]. Furthermore, previous studies have demonstrated that the two non-synonymous SNPs rs4986790/91 of *TLR4* might exhibit functional consequences at the cellular level and have relevance in genetic association studies, with contradictory findings [15].

Previous reports have demonstrated that both SNPs (rs4986790 and rs4986791) were in linkage disequilibrium among Caucasian populations [16] and co-segregate in the European populations (D’ = 1.0, *r*^2^ = 1.0) of both SNPs, and, thus, they could be studied together [17]. The co-segregation observed could be due to a drift caused by a genetic mutation of the *TLR4*D299G in Africa owing to the resistance to malarial infection [18].

Several research papers address the link of *TLR4* polymorphism with obesity and MeS [19,20], but such kind of studies among Arab subjects is scarce. The current study aimed at evaluating the associations within the non-synonymous missense mutations in the extracellular domain of the *TLR4* receptor polymorphisms, rs4986790 (ASP299GLY; 896A/G at exon 3) and rs4986791 (THR399ILE; 1196C/T at exon 3), with obesity and MeS components among the Arab female subjects.

## 2. Methods

### 2.1. Study Protocol

The present study was a prospective case-control association study. The study included 198 Arab female university students aged 19–30 years old. They were randomly recruited via advertising on the Qatar University campus and social media. The study was approved by the local committee of the institutional review board of human subjects at Qatar University (No. QU-IRB 682-EA/16) and follow the ethics and regulations of “the Declaration of Helsinki 2000”. After describing the purpose of the study, participants were requested to sign informed consent.

Subjects were excluded from the study if they had one of the following: pregnancy; cancer; liver, renal or cardiac disorders; inflammatory disorders; or anemia. Based on the BMI cut-off values, the study subjects were categorized into two groups, non-obese (control group) and obese (study group).

### 2.2. Study Design

Anthropometric measurements were performed using standard methods, and fasting blood samples were collected. Blood samples were utilized for DNA extraction and the biochemical assay. Furthermore, plasma was separated from the blood samples and used for measurements of the fasting blood glucose, lipid profile, insulin, hsCRP, leptin, IL-6 and Il-10. The experiments were performed in the Biomedical Labs at the College of Health Sciences (CHS), and Biomedical Research Center (BRC), Qatar University.

### 2.3. Anthropometrics Measurements

Following a standard procedure, the participant’s body weight was measured using an electronic scale (Seca) with accuracy to 0.1 kg (lightly dressed, without shoes). Standing height was measured using an electronic scale (Seca gmbh & co. kg). BMI was calculated by dividing the weight of the participant’s in kilograms over the height in meters squared. Waist circumference was measured while the participants were dressed in light clothes and a standing position. WC was used to categorize the study participants into two groups, standard (WC < 88 cm) and abnormally high (WC ≥ 88 cm). Body composition was evaluated using bioelectrical impedance analysis (BIA) using InBody 720 machine (Biospace Co.; Seoul South Korea). It measures total body water (TBW), fat mass (FM), fat-free mass (FFM) and percentage of body fat (PBF). Systolic and diastolic blood pressures were recorded in triplicate after resting by a standard sphygmomanometer in the seated position. The measurements were rounded to one decimal point, and all measures were evaluated in the nutrition lab (Department of Human Nutrition, CHS, Qatar University) by trained instructors [21].

### 2.4. Biochemical Assays

After overnight fasting, 10 mL of venous blood were collected from each participating subject: 5 mL were used for DNA extraction, and the remaining 5 mL were used for the measurement of plasma glucose, lipid profile, hsCRP, leptin and insulin. All biochemical measurements were performed at clinical chemistry labs at Biomedical Labs, Qatar University. hsCRP was measured in triplicate according to the manufacturer’s protocol provided by DRG, Germany (catalog No. EIA-3954) with an inter-assay CV of 5.2% and inter-assay CV of 6.3%. IL-10 was evaluated using MPXHCYTO-60K-06, and MULTIPLEX MAG Human Adipokine Magnetic Bead Panel 2 (MILLIPLEX^®^ MAP, catalog No. LHE-BD) kit was used for the quantification of the following biomarkers: IL-6, insulin and leptin based on the manufacturer’s protocol from Millipore (Merck Millipore, Bill-erica, MA, USA).

### 2.5. Genotyping of the Polymorphisms of the Variants rs4986790 and rs4986791

*TLR4* polymorphisms non-synonymous missenses mutations in the extracellular domain of the receptors rs4986790 (ASP299GLY; 896A/G at exon 3) and rs4986791 (THR399ILE; 1196C/T at exon 3) were carefully chosen for genotyping as previously published [18]. In short, DNA was extracted from the whole blood samples according to the protocol provided by the manufacturer (QiaAmp DNA blood Mini Kit Cat No. 51360). DNA concentration and purity were performed using the Nanodrop Spectrophotometer. The 5′ nuclease assay was used to determine the polymorphism of studied SNP by TaqMan MGB probe using an ABI 7500 (Applied Biosystems, Foster City, CA, USA). The primers and the probes of these SNPs polymorphisms were supplied by the assay-on demand TM service by Applied Biosystems. The 5′ nuclease assay was performed using 20 ng of genomic DNA, 1× TaqMan Universal PCR Master Mix (Applied Biosystems) and 1× primer/probe mix using the correct conditions for an extension by the manufacturer’s instructions. Negative controls were used as previously described [22].

### 2.6. Flowcytometry and Expression of TLR4 Receptors on Monocytes and Macrophages

Flow cytometry assay was utilized to measure the level of expression of TLR4 protein marker using CD 284 marker (BD-Biosciences) present on circulating monocytes of obese and non-obese subjects. Immunophenotyping was performed on the WBC, which was retrieved from the buffy coat. The buffy coat was isolated using Ficoll (Ficoll-PaqueTM PLUS, GE Healthcare Bio-sciences AB) from whole blood samples collected in tubes containing EDTA. *TLR4* monoclonal antibody (PE Mouse Anti-Human *TLR4*, CD 284 (BD PharmingenTM, BD-Biosciences) and stain buffer (FBS, BD PharmingenTM, BD-Biosciences) were added to tubes containing the extracted WBC. All tubes were analyzed using the flow cytometer BD LSRFortessa TM Cell Analyzer (Cat No. 649225). PE MOUSE IgG2K isotype control was used as a negative matched control. The data were analyzed using BD FACSDiva Software (BD Bioscience; San Jose, CA USA) software program. The data were expressed as mean fluorescence intensity (MFI) and/or percentage (%) of cells for the expression of CD 284 on monocytes. The monocyte population was identified by gating of CD14+HLA-DR+ cells using the size (forward scatter, FSC) and cell granularity as parameters (side scatter, SSC) where the cells presented in square gate P5 (Figures S1A and S2A) as described previously [23]. Unstained cells population was gated in square gate P2 (Figure S1B–D) and stained cells population was gated in square gate P1 (x > 102) (Figure S2B–D). For the technical analysis of flow cytometry with fasting samples, we included 21 obese subjects and 21 non-obese subjects, of different haplotypes.

### 2.7. Definitions and Cut-Off Values in the Study

The BMI cut-off values for this study were defined according to the WHO criteria into non-obese (BMI ≤ 30 kg/m^2^) and obese (BMI ≥ 30 kg/m^2^) [24]. Hypertension was defined as a systolic blood pressure ≥135 mmHg, diastolic blood pressure ≥85 mmHg, a significant history of hypertension and/or under-treatment of hypertension. HOMA was calculated based on the formula: “HOMA-IR = [glucose (nmol/L) × insulin (µU/mL)/22.5]”, using fasting values [25] and cut-off value for non-diabetic subjects [26]. MeS was determined according to the National Cholesterol, Education Program Adult Treatment Panel III [NCEP-III] [5]. The constellation of the MeS required the presence three components out of five of the following criteria: “hyperglycemia defined as fasting blood glucose ≥5.6 mM or 2 h blood glucose ≥11.1 mM measured during oral glucose tolerance test; dyslipidemia defined as fasting TG ≥1.70 mM or specific treatment for this lipid abnormality and/or HDL-C <1.29 for females; insulin resistance index by HOMA formula was ≥2.5 [27]; abdominal obesity defined by WC as >88 cm (>35 in) for women, and hypertension by ≥130/≥85 mmHg and/or on anti-hypertensive medications”.

### 2.8. Statistical Analysis

Data for outliers, skewness and normality were explored. All data are presented as mean ± SD for normally distributed variables unless stated otherwise. Categorical data such as frequencies of non-obese and obese subjects are given as numbers and percentages. Chi-square test assessed the differences between categorical variables. Genotype distributions and allele frequencies data are presented as number (n) and percentage (%) for genotype counting in all subjects. Minor allele frequency is portrayed as fractions and percentages and was analyzed using the chi-square test. In addition, the call rate of the genotyping and the Hardy–Weinberg equilibrium (HWE) was determined using the chi-square test to assess the distribution of genotypes in all study subjects. Logistic regression analysis was performed and odds ratio and 95% confidence intervals (Cs) were determined. The odds ratio was calculated using the homozygous minor allele as the reference group unless stated otherwise. Odds ratios were corrected for the Bonferroni test on SNP number and FDR. The data for the haplotype *TLR4*^D299G/T399I^ were analyzed by Golden Helix-Genetic data software. Two-tailed *p* value < 0.05 was considered as statistically significant. The statistical analyses were done using the SPSS program for Windows (IBM SPSS Statistics for Windows, Version 23.0. Armonk, NY, USA: IBM Corp). Graph Pad Prism was used for drawing the figures (version 8, for Win, Graph Pad Software, La Jolla, CA, USA). The Golden Helix SNP and Variation Suite (SVS 8, software; Bozeman, MT, USA) were used for the genetic analysis.

### 2.9. Power Calculations

We carried out a statistical power analysis using QUANTO software version 1.2.4 (http://hydra.usc.edu/gxe/_vti_bin/shtml.dll/request.htm) to guarantee our sample size was adequate to identify associations of examined SNPs and their susceptibility with obesity (40%). Under the population parameter settings, previous data indicated that MAF of rs4986791-G is 0.064 (NCBI Assay Id# ss86237807) and setting of the odds ratio of 2.5 and the dominant model of inheritance. We had to examine 178 subjects to be able to reject the null hypothesis that the rs4986791-G rates for cases and controls are equal with power of 0.8. The type I error probability associated with this test of this null hypothesis was 0.05.

## 3. Results

### 3.1. Phenotype Characteristics: Anthropometric and Biochemical Data of the Study Subjects 

The anthropometric and biochemical description of the study subjects is provided in Table 1. Both groups of the study, obese and non-obese, were age matched. The obese group had significantly higher mean values for the following variables compared to non-obese subjects: BMI, WC, %BF, TC, hsCRP, insulin, HOMA-IR and leptin (*p*-values < 0.0001, <0.0001, <0.0001, 0.032, 0.001, <0.0001, <0.0001 and 0.038, respectively). On the other hand, obese subjects had significantly lower HDL-C than non-obese subjects (*p*-value < 0.0001). No significant differences were detected between the two groups for age, systolic and diastolic blood pressure, glucose, TG and LDL-C.

### 3.2. TLR4 Genetic and Haplotype Distribution and Its Association with Obesity Phenotypes 

**Figure 1:**
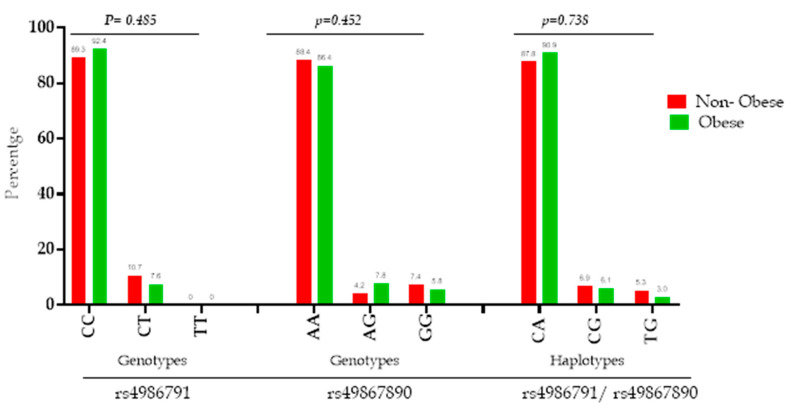
Genotypic and haplotype distribution in obese and non-obese subjects for the SNP rs4986790 (Asp299Gly) and SNP rs4986791 (Thr399Ile). Bars represent the genotype and haplotype distribution (%) of SNP rs4986790 and SNP rs4986791 among the study subjects. Two-tailed *p*-value is significant <0.05.

All SNPs reported a call rate >95%. In addition, both SNPs were tested in all study subjects using the Hardy–Weinberg equilibrium; only rs4986791 (Thr399Ile) is within the equilibrium (*p*-value = 0.477), while rs4986790 (Asp299Gly) shows a deviation from the Hardy–Weinberg equilibrium (*p*-value < 0.0001) (see Table S1). The results of this study were mainly based on the co-segregation of both SNPs, as they were in high linkage disequilibrium among different ethnic groups. Genotyping for both SNPs is presented in Table 2. For rs4986791 (Thr399Ile), the homozygous CC genotype has the predominant distribution in both non-obese and obese groups. The genotyping count and percentage for homozygous CC of rs4986791 in all subjects were (*n* = 178, 90.4%), in obese subjects (*n* = 61, 92.4%) and in non-obese subjects (*n* = 117, 89.3%). The minor allele frequency was T for Thr399Ile, with a frequency of 0.053 and 0.038 in non-obese and obese subjects (*p*-value = 0.493), respectively. For rs4986790 (Asp299Gly), the homozygous AA genotype has the predominant distribution in both the non-obese group and the obese group. The genotype AA of rs4986790 in all subjects is (*n* = 173, 87.4%), in obese subjects (*n* = 56, 86.4%) and in non-obese subjects (*n* = 117, 88.4%). The minor allele frequency is G for Asp299Gly, with a frequency of 0.098 and 0.090 in non-obese and obese subjects (*p*-value = 0.809), respectively. There were no significant differences in the genotype distribution between the two groups for both Asp299Gly and Thr399Ile (*p*-value = 0.452 and 0.485, respectively). Moreover, there was no minor homozygous (TT) for Thr399Ile. Figure 1 displays the genotype and haplotype distribution of both SNPs. Note that the frequency distribution in the percentage of CA haplotype was the most common in obese and non-obese subjects (90.9% and 87.8%, respectively); CG haplotype frequencies are 6.1% and 6.9% and TG haplotype frequencies are 3.0% and 5.3%, respectively. The distribution was not significantly associated with obesity with *p*-value = 0.738.

### 3.3. Linkage Disequilibrium (LD) of the Two SNPs (rs4986791 and rs4986790) in Study Subjects 

**Figure 2:**
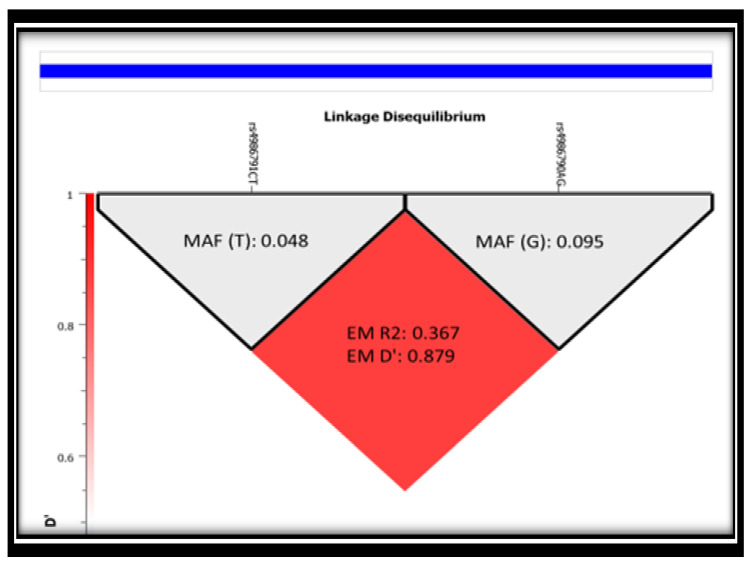
Linkage Disequilibrium (LD) of rs4986791 (Thr399Ile) and rs4986790 (Asp299Gly) in all study subjects. The red diamond represents pairwise LD expressed as EM R2 and EM D values between rs4986791 and rs4986790, respectively. The grey triangles represent minor allele frequency (MAF) of each genetic variant in all study subjects.

Pairwise LD between the two polymorphisms was determined using data from all subjects. Using expectation maximization (EM), the results of LD for all study subjects are presented in Figure 2. The results of the LD of the two SNPs had strong LD (0.879) in the study subjects. Therefore, all SNPs rs4986791 and rs4986790 were incorporated in the analysis and were strongly inherited with each other and could be studied as haplotype polymorphism. The *r*^2^ value of 0.367 was caused by the difference in the frequency of the minor alleles of both SNPs, and each SNP could not substitute the other (Figure 2). The haplotypes formed between two SNPs were three pairs (CA, CG and TG), as shown in Figure 1, due to missing rare homozygous alleles TT in rs4986791.

### 3.4. The Frequency Distribution of the Metabolic Syndrome among the Study Subjects 

**Figure 3:**
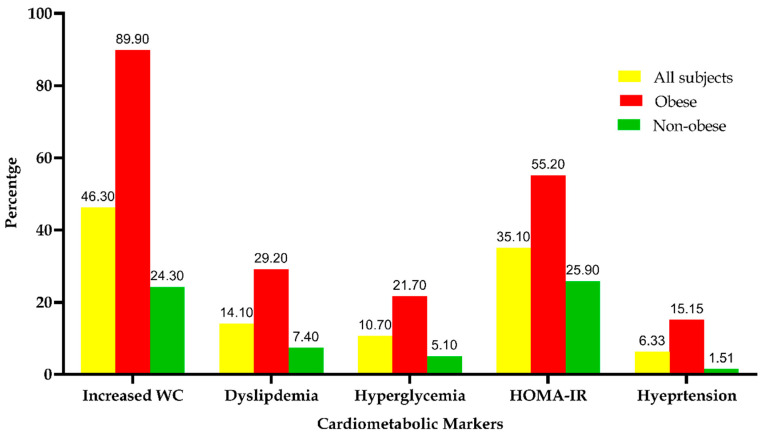
Frequency of metabolic syndrome components among study subjects based on obesity status. Bars represent the frequency distribution (%) of metabolic syndrome components among all study subjects, obese subjects and non-obese subjects based on BMI cut-off values.

Figure 3 displays the frequency distribution of MeS components among the study population. Obese subjects compared to non-obese subjects had a significant increase in WC (89% vs. 24.30%), dyslipidemia (29.2% vs. 7.40%), hyperglycemia (2.70% vs. 5.10%), HOMA-IR (55.20% vs. 25.90%) and hypertension (15.15% vs. 1.51%) with *p*-values < 0.0001.

### 3.5. Association between Obesity Phenotypes and Dominant Genetic Models of rs4986790 and SNP rs4986791 among the Study Subjects 

**Figure 4:**
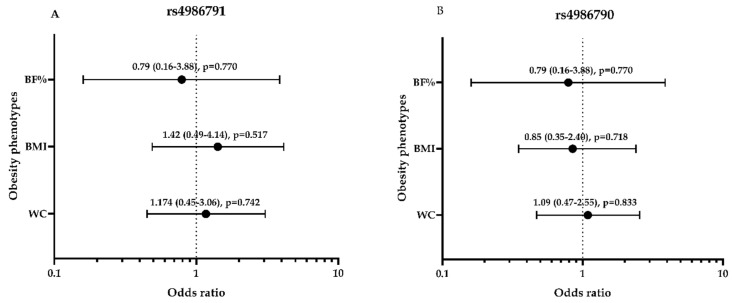
The genetic variants rs4986791 (Thr399Ile) and rs4986790 (Asp299Gly) polymorphisms-related odds ratios and 95% CI with obesity phenotypes. Data are expressed as odds ratio (odds ratio of 95% lower and upper confidence interval (CI) with their *p*-values) for the independent variables of obesity phenotypes: %BF, BMI and WC with rs4986791 (**A**) and rs4986790 (**B**) in their dominant model in obese subjects versus non-obese as controls. Logistic regression analysis was used for data analysis. Two-tailed *p*-value is significant at ≤0.05. BMI, Body Mass Index; WC, Waist Circumference; BF%, Body Fat percentage.

Furthermore, we used the regression analysis to evaluate the odds ratio with the polymorphisms of individual SNPs utilizing the dominant genetic model with obesity by the adiposity phenotypes BF%, BMI and WC categories among the study subjects. As displayed in Figure 4, the results demonstrate no significant association of the genetic variants Thr399Ile and Asp299Gly with the risk of getting obesity using different obesity phenotypes (BF%, BMI and WC) with the *p*-value > 0.05. The mean values of BMI, WC and %BF levels based on the genotype distribution of SNP rs4986790 and SNP rs4986791 among the study subjects were evaluated, as presented in Table S2. There were no significant differences in the mean values of WC, BMI, and BF% for rs4986791 (*p* = 0.741, 0.955 and 0.423, respectively). In addition, there was no significant difference in the mean values of rs4986790 with WC, BMI, and %BF with (*p* = 0.942, 0.185 and 0.722, respectively).

Further, we utilized the regression analysis to evaluate the association between obesity as the dependent variable and the independent variables (WC, lipid profile and HOMA). The following independent variables were included in this analysis: WC, TC, HDL, LDL and HOMA. As indicated in Table S3, WC and HOMA -IR demonstrated significant associations with obesity (OR: 53.19, 95% CI (9.12, 310.31); and OR: 3.52, 95% CI 1.90, 6.53, respectively; *p* < 0.0001). TC, HDL and LDL showed no significant association with obesity (OR: 0.259, 95% CI: 1.425, 1.083, *p* > 0.05).

### 3.6. rs4986791 and rs4986790 Polymorphisms-Related Odds Ratios with MS Components and Lipid Profile 

**Figure 5:**
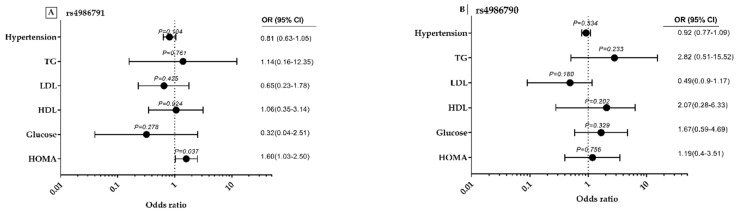
The genetic variants rs4986791 (Thr399Ile) and rs4986790 (Asp299Gly) polymorphisms-related odds ratios and (95% CI) with metabolic syndrome components and lipid profile. Data are expressed as odds ratio (odds ratio of 95% lower and upper confidence interval (CI) with their *p*-values) for the effect of the following metabolic risk factors: hypertension, hyperglycemia, HOMA-insulin resistance and lipid profiles with each genetic variant (in their dominant model) in obese subjects versus non-obese as controls for rs4986791 (panel **A**) and for rs4986790 (panel **B**). Logistic regression analysis was used for data analysis. Two-tailed *p*-value is significant <0.05. TC, Total Cholesterol; TG, Triglycerides; HDL-C, High Density Lipoprotein; LDL-C, Low Density Lipoprotein.

As indicated in Figure 5A,B, regression analysis was applied to assess the interaction between the variables using the dominant genetic model of SNP rs4986791 and SNP rs49867901 with the independent variables of MeS components and the lipid profile among obese subjects. The following MeS components were included in this analysis: HOMA, glucose, HDL, TG and blood pressure. As indicated in Figure 4A, rs4986791 polymorphism significantly increased the risk of getting insulin resistance (HOMA) with an OR of 1.60 and 95% CI of 1.03–2.50, with *p* = 0.037. TG, HDL, LDL, glucose and blood pressure showed no significant association with obesity. Furthermore, as indicated in Figure 5B, the following MeS components did not show any significant association with obesity (*p*-value > 0.05): HOMA, TG, HDL, LDL, glucose and blood pressure.

Mean values of MeS components and Lipid profile based on the genotype distribution of SNP rs4986790 and SNP rs4986791 using the dominant genetic model for subjects are presented in Tables S4 and S5. Individuals carrying the CT for the SNP rs4986791 had significantly higher insulin and HOMA mean values in comparison to individuals carrying the homozygous major allele CC. Additionally, for both polymorphisms, there was no significant association with other metabolic risk markers, namely TC, HDL, LDL, glucose and blood pressure.

### 3.7. Haplotype Frequency and Its Association with Obesity, WC, HOMA and Dyslipidemia 

**Figure 6:**
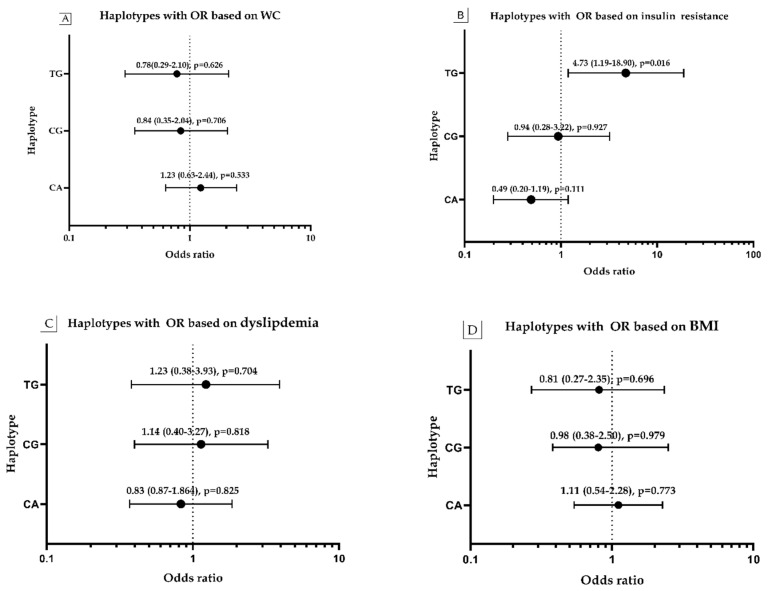
Haplotype association tests of co-segregated rs4986791 C > T, rs4986790 A > G with WC, IR, dyslipidemia, and BMI among obese subjects compared to non-obese subjects. Data are expressed as odds ratio (odds ratio of 95% lower and upper confidence interval (CI) with their *p*-values) for the independent variables: waist circumference (**A**); HOMA-insulin resistance (**B**); dyslipidemia (**C**); and BMI (**D**) with the haplotypes (CA, CG and TG) of the rs4986791 C > T and rs4986790 A > G among obese versus non-obese as controls. Logistic regression analysis was used for data analysis. Two-tailed *p* value is significant <0.05.

As indicated in Tables S6–S10 and displayed in Figure 6A–D, the alleles of the SNPs rs4986790 A > G and rs4986791 C > T, located in chromosomal locus chromosome 9q32–q33, established several frequent haplotypes with their associations with metabolic risk markers of cardiometabolic syndrome. As indicated in Figure 6B, only the TG haplotype formed by the rare alleles of the studied SNPs had a significant effect on the risk of insulin resistance (HOMA) in obese subjects compared to non-obese subjects with odds of 4.73 times (95% CI 1.19–18.90, *p* = 0.016). The frequency of IR in obese subjects is nearly 4.58 times (87.0% vs. 19.0%, *p* = 0.0165) its frequency in non-obese (Table S7). In addition, the other haplotypes formed have an insignificant effect on the risk of obesity by WC (Table S6 and Figure 6A), dyslipidemia (Table S8 and Figure 6C), BMI (Table S9 and Figure 6D) and hyperglycemia (Table S10).

### 3.8. The Expression of CD284 of TLR4 in Study Subjects by Flow Cytometry

Table 3 displays the protein expression of *TLR4* (CD284) as Mean Fluorescent Intensity (MFI) in non-obese and obese subjects. The data demonstrate a significantly higher concentration of TLR4 protein expression in obese subjects compared to non-obese subjects (*p* < 0.0001). Table 3 indicates that the obese subjects had significantly higher IL-6 concentration (12.42 ± 3.41 pg/mL) than the non-obese subjects (1.31 ± 0.35 pg/mL). Circulating IL-10 was significantly lower in obese (2.56 ± 0.52 pg/mL) than non-obese subjects (3.91 ± 0.64 pg/mL).

### 3.9. Association between CD284 of TLR4 and the Genotype and Haplotype Distribution of Two SNPs (rs4986791 and rs4986790)

To evaluate the relation between *TRL4* expression and haplotype/genotype, we evaluated the association between genotype and haplotype distribution in obese and non-obese subjects (Table 4). For rs4986791 (Thr399Ile), the homozygous CC genotype has the predominant distribution in both non-obese and obese groups. The genotyping count and percentage for homozygous CC of rs4986791 in all subjects were (*n* = 38, 90.5%), in obese subjects (*n* = 19, 90.5%) and in non-obese subjects (*n* = 19, 90.5%). CT genotype distribution were similar in obese and non-obese groups at 10.5%, and the distribution is not significant (*p*-value = 1.00). For rs4986790, AA genotype is predominant in non-obese (17, 80.9%) and in obese (18, 85.7%). AG genotype was found in one (4.8%) and two (9.5%) while GG genotype in three (14.3%) and one (4.8%) non-obese and obese subjects, respectively, and the distribution was not significant with *p*-value of 0.506. Further, we evaluated the haplotype distribution among the selected cohort used to study TLR expression. As shown in Table 5, no significant association of the haplotypes AA, CG and TG and its distribution among the obese and non-obese subjects with *p*-value of 0.834 was found.

Furthermore, we quantified the MFI of TLR expression in all subjects of the cohort and, based on the interquartile distribution, we selected two groups of low expression of ≤25% (cut off value of 2600 MFI) and high expression of ≥75% (cut off value of 3571 MFI) to study the association with genotype and haplotype distribution, as shown in Table 5. Further, the data demonstrate no significant association for genotypes of rs4986791 (*p* = 0.486) and rs4986790 (*p* = 0.281), as well as for the haplotypes of rs4986791 and rs4986790 (*p* = 0.765).

### 3.10. The Correlation between Haplotype of Two SNPs (rs4986791 and rs4986790) and IL6, IL10, and CD284 of Monocyte TLR4 Expression 

Further, we evaluated the Pearson coefficient correlation (*r*) between the haplotypes of rs4986791 and rs4986790 among the cohort of the study subjects for whom the TLR4 expression was evaluated. As displayed in Table 6, the haplotype correlations coefficient (*r*) with TLR4, IL-6 and, IL-10 are not significant (*p*-values ≥ 0.05). TLR4 expression was significantly correlated directly with IL-6 (*p* < 0.0001) and inversely significantly correlated with IL-10 (*p* < 0.0001). IL-6 was inversely significantly correlated with IL-10 (*p* < 0.0001).

## 4. Discussion

The current study explored the impact of *TLR4* receptor genotypes rs4986790 and rs4986791 polymorphisms (*TLR4*^D299G/T399^) and TLR4 protein expression in obesity and metabolic syndrome components. BMI, WC, lipid profile, glucose, insulin and HOMA were used as surrogates of obesity and MeS indicators in the current study. The results of the present study demonstrate two key findings. First, there was a significant association between the haplotype of rare alleles (TG) of *TLR4*^D299G/T399^ polymorphism with HOMA in obese subjects, which is the dominant feature of MeS. Second, a significant upregulation of TLR4 protein expression was detected in obese subjects. No significant associations with other components of the metabolic syndrome, glucose, TG, HDL, WC and blood pressure were noted in the current study with *TLR4*^D299G/T399^ polymorphism.

The current study reported a strong LD of both polymorphisms Asp299Gly (rs4986790) and Thr399Ile (rs4986791) with D’ = 0.879. In support of the current finding, a prior study by Santos et al. (2006) demonstrated that Asp299Gly (rs4986790) and Thr399Ile (rs4986791) polymorphisms were in linkage disequilibrium, D’ = 0.88 [28]. Additionally, previous studies confirmed the co-segregation of rs4986790 (exon 3, D299G) with rs4986791 (exon3, T399I) and their linkage disequilibrium [20]. In the current study among Arab female subjects, the minor allele frequencies of rs4986791(C > T) and rs4986790 (A > G) were similar to the global minor allele frequencies (Table S6) retrieved from the Reference SNP (rs) Report by the National Center for Biotechnology Information (NCBI), (https://www.ncbi.nlm.nih.gov/snp/rs4986791 and https://www.ncbi.nlm.nih.gov/snp/rs4986790).

The TLR family includes pathogen-specific receptors expressed and present in leukocytes and other cells, including hepatocytes and adipocytes. Both adipose tissue macrophages and adipocytes express TLR on their plasma membranes and interact to produce several pro-inflammatory chemokines and cytokines due to their close anatomical site in adipose tissue [29]. In addition, *TLR4* contributes immensely to the pathogen identification and stimulation of the human immune system. A previous study reported that innate immunity modulated by *TLR4* and the intestinal microbiota could be the missing connection in the MeS pathogenesis which link obesity to diabetes mellitus [30].

The activation of *TLR4* may contribute to MeS, insulin resistance and endoplasmic reticulum stress. The present study did not find any association of D299G/T399I haplotypes with obesity phenotype per se indicators, namely BMI, WC and %BF. Furthermore, the current data detect a significant association of T allele carriers of Thr399Ile and haplotype TG of D299G/T399I with insulin resistance. Our findings are endorsed by previous studies with clinical and experimental evidence, implying that *TLR4* receptor genotypes rs4986790 and rs4986791 polymorphisms may be linked to the marked inflammation and upregulation of the receptor which is linked to insulin resistance and pathogenesis of diabetes [31]. *TLR4* activation triggers the upstream regulator of inflammatory pathways linked to the generation of insulin resistance, such as SOCS3, NF-κB and JNK [32]. A study by Ran Ao et al. (2015) reported that *TLR4* receptor genotypes rs4986790 and rs4986791 polymorphism are associated with marked inflammation [33].

Furthermore, this finding was also verified by the study of Yang Cheng et al. (2016), which reported that *TLR4*^D299G^ *TLR4*^T399I^ was associated with an increased risk of inflammatory bowel disease [34]. A study by Weyrich P et al. (2010) reported that heterozygous carriers of rs4986790 polymorphism were associated with a substantial increase in liver fat and visceral adipose tissue [20], which support the association between *TLR4* polymorphism and inflammatory mediated obesity. In contrast to the previous studies, few studies have indicated that *TLR4*^D299G^ *TLR4*^T399I^ was related to decreased inflammation and the risk of MeS. A previous study by Penas-Steinhardt A et al. (2012) reported that *TLR4* + 3725G/C polymorphism in the 3′UTR might be associated with lower prevalence (protective) of overweight and chronic metabolic disorders [35]. The data of the current study demonstrate an increase in CRP and IL-6 levels in obese subjects, which could highlight the marked inflammatory process that is linked to insulin resistance in obese subjects. A previous study demonstrated that Asp299 allele is associated with T2D, which is always preceded by inflammatory changes, with the development of MeS and IR [36]. A previous study indicated a high association of *TLR4* haplotypes, which comprise rs4986791 (Thr399Ile) polymorphisms, with the risk of T2D [37]. In our study, Thr399Ile was in high linkage disequilibrium with Asp299Gly. These data provide evidence of our results that a *TLR4*^D299G/T399^ variant could impact on IR of obese subjects.

In addition, the current study demonstrated the presence of a significant association between *TLR4*^D299G/T399I^ polymorphism with insulin resistance. Individuals carrying the CT genotype for the SNP (rs4986791) had substantially higher levels of insulin and resistance to insulin. In addition, *TLR4*^T399I^ demonstrated a significant association with the risk of increased insulin resistance with an OR of 1.60 and *p* = 0.037. These data indicate that the *TLR4*^T399I^ polymorphism was associated with increased IR, which is a substantial risk factor of MeS and diabetes. Arbour et al. (2000) were the first to demonstrate that subjects with either the Asp^299Gly^ and/or Thr^399Ile^ polymorphisms had a dampened response to LPS in experiments [14]. This co-segregated state of *TLR4* indicated four characteristic haplotypes in the population: wt/wt, Asp299Gly/wt, Thr399Ile/wt and Asp299Gly/Thr399Ile [14]. In the current study, Asp299Gly was not associated with IR itself; however, it was tagged by Thr399Ile, which existed in high linkage disequilibrium with Asp299Gly. Additionally, the TG haplotype of *TLR4*^D299G/T399I^ constructed by the minor alleles of the investigated polymorphisms Asp299Gly/Thr399Ile had a remarkable impact on the risk of the IR assessed by HOMA with odd of 4.73 and *p*-value of 0.016. This finding is supported by a previous study which stated that *TLR4*^D299G/T399I^ was associated with an increase in IR in non-diabetic patients [20]. These two SNPs (rs4986790 and rs4986791) were associated with insulin resistance as a critical factor of the metabolic syndrome and lipid accumulation in Argentinian populations [17].

Further, the present study demonstrated a significant upregulation of the cell surface protein expression of *TLR4* receptors in monocytes of the obese subjects. The current study demonstrated a significant increase in IL-6 with a concomitant decrease of IL-10, which indicate a pro-inflammatory state. Previous studies reported upregulated expression of TLR4 protein in PBMCs of obese subjects and allied with increased manufacture of pro-inflammatory markers of TNF-α and IL-6 [38]. A study by Song et al. in 2006 showed that activation of *TLR4* in the adipocyte was involved in the inception of insulin resistance in obesity and type 2 diabetes. [39]. A previous study revealed that TLR4 protein expression is upregulated on visceral adipose tissue-derived macrophages from obese subjects, which was related to the expansion in adipocyte size and increased CRP levels [40]. Furthermore, in support of the findings of the present study, previous studies with a knockout of *TLR4* gene in mice resulted in partial defense against the development of IR after lipid infusion or high-fat diet [41,42].

The increased expression of *TLR4* in the obese compared to the non-obese subjects may be due to increased *TLR4* ligands, free fatty acids and lipopolysaccharide (FFA and LPS) in the obese patients. As reported in a prior study by Velloso LA et al. (2015), *TLR4* could be triggered by LPS and free fatty acids (FFA) released from the gut due to the weakening of gut integrity caused by obesity and other factors [43]. Additionally, a study by Vitseva, O. I. et al. (2008) reported that *TLR4* activity might have a significant role in obesity-associated inflammation and development of cardiometabolic risk in adipose tissues [44]. The overexpression of muscle *TLR4* has been described in subjects with insulin resistance [45]. In the current study, obese subjects exhibited an increased WC, indicative of increased visceral fat that is associated with leptin and IR. This, in turn, could enhance the lipolysis and the release of FFA [46] that may act as a *TLR4* ligand, causing inflammation and β-cell dysfunction [47]. Thus, *TLR4* could be the missing connection among inflammation, IR and obesity.

The current study revealed that the upregulation of *TLR4* in obese subjects with marked IR was associated with the haplotype polymorphism of co-segregated *TLR4* D299G/T399I. *TLR4* polymorphism D299G/T399I present on exon 3, which could affect the transmembrane and hypervariable segment of the *TLR4* receptor. Such polymorphism, in theory, could impair the ligand binding and its activity for signaling mechanisms in adipose tissue cells to activate the transcription factor NF-kB. It acts as an upstream regulator affecting the mRNA expression of pro-inflammatory cytokines such as TNF and IL-6 released by the adipocytes [12]. In contrast, a previous study using stably transfected cell lines of 293/hMD2-CD14 disclosed that the *TLR4* polymorphism D299G/T399I did not change the protein expression of *TLR4* and its subcellular locations. Moreover, in that study, the Asp299Gly, but not Thr399Ile, polymorphism reduced the response of the immune system of *TLR4*, as evaluated by the production of pro-inflammatory cytokine and the stimulatory effect of NF-κB in response to LPS [48]. The current data demonstrate that *TLR4*D299G/T399I haplotype polymorphism is not significantly associated with monocytes TLR protein expression (Table 5; Table 6). Moreover, the pro-inflammatory markers IL-10 and IL-6 are not significantly correlated with *TLR4*D299G/T399I polymorphism (Table 6). This study is in line with our findings that *TLR4* Asp299Gly and Thr399Ile polymorphisms did not affect the expression level of TLR4 protein and the inflammatory markers of CRP, IL-6 and IL-10 in obese subjects. Further studies should address the *TLR4* signaling in adipocyte driven from *TLR4*^D299G/T399I^ carriers. In the present study, we used monocytes to evaluate TLR4 protein expression, as previously authors have used monocytes *TLR4* as one of the biomarkers to characterize metabolic syndrome in obese subjects [29,39,49,50], as visceral adipose tissue was not feasible to obtain for human adipocytes from the study subjects.

In conclusion, carriers of TC haplotype of *TLR4*^D299G/T399I^ were associated with IR evaluated by (HOMA-IR) in obese individuals that may contribute to the risk of MeS obese subjects. *TLR4*^D299G/T399I^ polymorphism had no impact on TLR4 protein expression or the pro-inflammatory IL-6 and IL-10 in the study subjects. Increased protein expression of TLR4 protein in monocytes of WBC in obese subjects highlighted the role of *TLR4* as a connection among inflammation, IR and obesity. Therefore, subjects with TC haplotype carriers and with upregulated TLR4 protein expression are at increased risk for the pathogenesis of insulin resistance and MeS in obese subjects.

The current study had several limitations, including the limited sample size (198 subjects) and the gender of the population (Arab female subjects only). Besides, only two SNP in the *TLR4* were studied. Further studies are needed on larger sample sizes, with more SNPs of *TLR4*, various ages and both genders included. In addition, evaluation of gut microbiome and free fatty acid levels are needed to study its associations with *TLR4*D299G/T399I haplotype polymorphism.

## 5. Plain Summary

*TLR4*^D299G/T399I^ polymorphism among haplotype GT carriers is associated with increased insulin resistance in obese subjects, a core component of MeS.

Increased cell surface protein expression of *TLR4* in PMBC is detected in obese subjects, including haplotype GT carriers.

The upregulation of TLR4 protein plus the polymorphism of *TLR4*^D299G/T399I^ may highlight the higher risk and the impact of *TLR4* in obesity-associated inflammation with IR that precede diabetic and cardiovascular events.

The hyperleptinemia associated with insulin resistance could play a role in the hydrolysis of fat to free fatty acids that act as a ligand for *TLR4*.

The present study demonstrated no association of *TLR4*^D299G/T399I^ with obesity per se indicators, namely BMI, WC and %BF.

*TLR4* is a molecular link among the immune system, inflammation, IR and obesity.

Obese subjects with haplotype GT carriers of *TLR4*^D299G/T399I^ are at a higher risk for comorbidities associated with obesity such as MeS, IR and T2DM.

## Figures and Tables

**Table 1:** The anthropometric measurements and biochemical markers in non-obese subjects compared to obese subjects based on waist circumference.

Variables	NON-Obese (*n* = 132)	Obese (*n* = 66)	*p*-Value
Age (years)	22.04 ± 3.39	21.45 ± 2.67	0.215
BMI (kg/m^2^)	22.72 ± 3.60	35.65 ± 4.01	<0.0001
WC (cm)	80.26 ± 9.01	102.88 ± 8.22	<0.0001
%BF	33.91 ± 7.04	48.06 ± 3.43	<0.0001
Systolic blood pressure (mmHg)	114.24 ± 6.10	121.27 ± 8.40	0.785
Diastolic blood pressure (mmHg)	72.25 ± 4.45	78.55 ± 7.85	0.425
Glucose (mmol/L)	4.95 ± 0.43	5.06 ± 0.71	0.251
TC (mmol/L)	3.95 ± 0.26	4.03 ± 0.93	0.032
TG (mmol/L)	0.79 ± 0.37	0.93 ± 0.68	0.121
HDL-C (mmol/L)	1.37 ± 0.36	0.90 ± 0.39	<0.0001
LDL-C (mmol/L)	2.35 ± 0.80	2.19 ± 1.59	0.386
hsCRP (pg/mL)	1.19 (0.21–4.01)	4.94 (1.66–7.78)	0.001
Insulin (μU/L)	10.68 (4.71–15.54)	16.05 (5.50–19.25)	<0.0001
HOMA (insulin resistance) (IR)	1.83 (1.08–3.53)	3.67 (1.87–4.74)	<0.0001
Leptin (ng/mL)	8.19 (3.36–13.12)	20.25 (17.18–37.82)	0.038

Data are presented as mean value ± SD. Data were analyzed using unpaired student’s *t*-test. Two-tailed *p*-value is significant at <0.05. BMI, Body Mass Index; WC, Waist Circumference; BF%, Body Fat percentage; TC, Total cholesterol; TG, Triglycerides; HDL-C, High density lipoprotein; LDL-C, Low density lipoprotein; hsCRP, High-sensitivity C-reactive protein.

**Table 2:** Genotype distribution and minor allele frequency in non-obese group versus obese group.

**SNP**	**Genotype**	**All Subjects** **(*n* = 197)**	**NON-Obese** **(*n* = 131)**	**Obese** **(*n* = 66)**	***p*-Value**
rs4986791 (+1196 C > T) Thr399Ile	CC	178 (90.4%)	117 (89.3%)	61 (92.4%)	0.485
	CT	19 (9.6%)	14 (10.7%)	5 (7.6%)	
Minor allele frequency (T)		0.048	0.053	0.038	0.493
**SNP**	**Genotype**	**All Subjects** **(*n* = 198)**	**NON-Obese** **(*n* = 132)**	**Obese** **(*n* = 66)**	***p*-Value**
rs4986790 (+896 A > G) Asp299Gly	AA	173 (87.4%)	117 (88.4%)	56 (86.4%)	0.452
	AG	12 (6%)	4 (4.2%)	8 (7.8%)	
	GG	13 (6.6%)	11(7.4%)	2 (5.8%)	
Minor allele frequency (G)		0.096	0.098	0.090	0.809

Data are presented as number (n) and percentage (%) for genotype counting in all subjects (non-Obese group and obese group). Minor allele frequency is portrayed as a fraction. Data were analyzed using a chi-square test. Two-tailed *p*-value is significant at ≤0.05.

**Table 3:** The cell surface expression of monocytes *TLR4* expressed as mean fluorescent intensity (MFI), IL-6 and IL-10, in non-obese versus obese subjects.

Variables	Non-Obese (*n* = 21)	Obese (*n* = 21)	*p*-Value
*TLR4* Mean Fluorescent Intensity (MFI)	2536.00 ± 641.60	3444.00 ± 461.30	<0.0001
IL-6 (pg/mL)	1.31 ± 0.35	12.42 ± 3.41	<0.0001
IL-10 (pg/mL)	3.91 ± 0.64	2.56 ± 0.52	0.045

Data are presented as mean ± SD. Data are expressed as arbitrary units (MFI) for flow cytometry in Arbitrary Units (AU). Two-tailed *p*-value is significant at <0.05.

**Table 4:** Genotype and haplotype distribution of rs4986791 and rs4986790 in the cohort of non-obese group versus obese subjects included in monocyte TLR4 expression analysis.

**SNP**	**Genotype**	**All Subjects** **(*n* = 42)**	**Non-Obese** **(*n* = 21)**	**Obese** **(*n* = 21)**	***p*-Value**
rs4986791 (+1196C > T) Thr399Ile	CC	38(90.5%)	19(90.5%)	19 (90.5%)	1.000
	CT	4 (9.5%)	2 (10.5%)	2 (10.5%)	
**SNP**	**Genotype**	**All Subjects** **(*n* = 42)**	**Non-Obese** **(*n* = 21)**	**Obese** **(*n* = 21)**	***p*-Value**
rs4986790 (+896A > G) Asp299Gly	AA	35 (83.3%)	17 (80.9%)	18 (85.7%)	0.506
	AG	3 (7.1%)	1 (4.8%)	2 (9.5%)	
	GG	4 (9.5%)	3 (14.3%)	1 (4.8%)	
	**Haplotype**	**All Subjects** **(*n* = 42)**	**Non-Obese** **(*n* = 21)**	**Obese** **(*n* = 21)**	***p*-Value**
rs4986790 (+896A > G) Asp299Gly	CA	35 (83.3%)	17 (80.9%)	18 (85.7%)	0.834
	CG	3 (7.1%)	2 (9.5%)	1(4.8%)	
	TG	4 (9.5%)	2 (9.5%)	2(9.5%)	

Data are presented as number (n) and percentage (%) for genotype and haplotype counting in the cohort (non-obese group and obese group). Data were analyzed using a chi-square test. Two-tailed *p*-value is significant at ≤0.05.

**Table 5:** Genotype and haplotype distribution of rs4986791 and rs4986790 among subjects with low monocyte TLR4 expression (≥25%) and high monocyte TLR4 expression (≥75%).

Genotype	Monocyte CDTLR4 Expression		
**rs4986791**	**Low expression group** **(≥25%) (*n* = 11)**	**High expression group** **(≥75%) (*n* = 10)**	***p*-Value**
CC	10 (90.9%)	8 (80.0%)	0.486
CT	1(9.1%)	2 (20.0%)	
**rs4986790**			
AA	9 (81.8%)	7 (70.0%)	0.281
AG	0 (0.0%)	2 (20.0%)	
GG	2 (18.2%)	1 (10.0%)	
**Haplotype** **rs4986790 &** **rs4986790**	**Low expression group** **(≥25%) (*n* = 11)**	**High expression group** **(≥75%) (*n* = 10)**	***p*-Value**
CA	9 (81.8%)	7 (70.0%)	0.765
AG	1 (9.1%)	1 (10.0%)	
TG	1 (9.1%)	2 (20.0%)	

Data are presented as number (n) and percentage (%) for haplotype counting in the cohort (non-obese group and obese group). Data were analyzed using a chi-square test. Two-tailed *p*-value is significant at ≤0.05.

**Table 6:** Pearson coefficient correlation (r) between haplotypes rs4986790 and rs4986791 and IL-6, IL-10 and CD284 of monocyte TLR4 among the cohort of study subjects.

	IL-6	IL-10	TLR4	Haplotype
Variables	*r* *p*	*r* *p*	*r* *p*	*r* *p*
IL-6	1	−0.675 <0.0001	0.595 <0.0001	0.036 0.819
IL-10	−0.675 <0.0001	1	−0.654 <0.0001	0.009 0.095
*TLR4*	0.595 <0.0001	−0.654 <0.0001	1	0.324 0.0598
Haplotype	0.036 0.819	0.009 0.095	0.324 0.0598	1

Two-tailed *p*-value is significant <0.05.

## Supplementary Materials

The following are available online at https://www.mdpi.com/2073-4425/11/7/814/s1. Table S1: The call rate and HWE for all study subjects in rs4986790 and 4986791; Table S2: The mean values of BMI, WC and % BF levels based on the genotype distribution of SNP rs4986790 and SNP rs4986791 among the study subjects; Table S3: The association between obesity as the dependent variable with the following independent variables (WC, TC, HDL, LDL, and HOMA); Table S4: Mean values of MS markers (WC, Insulin, HOMA, Glucose, and Blood Pressure) among study subjects based on the genotype; Table S5: Mean values of lipid profile (TC, TG, HDL, LDL) among the study subjects based on the genotype; Table S6: Estimated haplotype frequency of the co-segregated rs4986791 C>T, and rs4986790 A>G related odds ratios associated with WC as obesity phenotype; Table S7. Estimated haplotype frequency of the co-segregated rs4986791 C>T, and rs4986790 A>G related odds ratios associated with HOMA-IR as MS component; Table S8: Estimated haplotype frequency of the co-segregated rs4986791 C>T, and rs4986790 A>G related odds ratios associated with Dyslipidemia; Table S9: Estimated haplotype frequency of the co-segregated rs4986791 C>T, and rs4986790 A>G related odds ratios associated with BMI as obesity phenotype; Table S10. Estimated haplotype frequency of the co-segregated rs4986791 C>T, and rs4986790 A>G related odds ratios associated with hyperglycemia; Table S11: Genotype distribution of TLR4D299G and TLR4T399I in different populations; Table S12: Monocyte cell surface markers used in the study; Figure S1: The gating steps for expression of CD284 on Monocytes (unstained cells); Figure S2: The gating steps for expression of CD284 on Monocytes (stained cells).
