# Supplementary material for: TLR4 Receptor D299G/T399I Haplotype Polymorphism Is Associated with Insulin Resistance in Obese Female Subjects
Source: Genes (Basel). 2020 Jul 17;11(7):814. doi: 10.3390/genes11070814 (PMC7397302; doi:10.3390/genes11070814)
Supplement: Supplementary file 1 [file genes-11-00814-s001.pdf]

Supplementary Data

Supplementary tables

Table S1. The call rate and HWE for all study subjects in rs4986790 and 4986791

TLR 4 SNP	rs4986790 (A>G)	rs4986791 (C>T)
**Call rate**	96%	96%
**Hardy-Weinberg equilibrium**	<0.0001	0.477

Call rate is presented as (%) of successful genotype to all samples performed. HWE is calculated by the Chi-square test.

Table S2. The mean values of BMI, WC and % BF levels based on the genotype distribution of SNP rs4986790 and SNP rs4986791 among the study subjects.

Genotype/SNP	*WC (cm)*	*BMI (kg/m²)*	*%BF*
		**rs4986791 C>T**	
**CC**	86.10±13.56	27.21±7.24	35.91±8.37
**CT**	85.00±11.04	25.80±6.27	34.11±7.07
P value	0.741	0.955	0.423
		**rs4986790 A>G**	
**AA**	86.03±13.20	26.92±6.99	36.25±8.32
**AG**	87.60±14.18	29.66±8.67	33.74±7.81
**GG**	86.23±11.96	24.46±5.68	35.04±8.22
P value	0.942	0.185	0.722

Data are presented as mean and SD of WC (cm), BMI (kg/m²), and %BF among all study subjects. Data are analyzed by unpaired Student t-test. Two-tailed *P*-value is significant at ≤ 0.05.

Table S3. The association between obesity as the dependent variable with the following independent variables (WC, TC, HDL, LDL, and HOMA)-

Variables	Odds Ratio (OR)	95% OR (lower confidence bound)	95% OR (upper confidence bound)	*P*-value
**WC**	53.19	9.117	310.307	<0.0001
**TC**	0.259	0.163	9.748	0.826
**HDL**	1.425	0.210	9.677	0.717
**LDL**	1.083	0.208	5.182	0.963
**HOMA-IR**	3.52	1.90	6.53	<0.0001

Data are expressed as odds ratio (odds ratio of 95% lower and upper confidence interval (CI) with their *P*-values) for the independent variables among obese versus non-obese as controls. Logistic regression analysis is used for data analysis. Two-tailed *P*-value is significant at ≤ 0.05.

Table S4. Mean values of MS markers (WC, Insulin, HOMA, Glucose, and Blood Pressure) among study subjects based on the genotype.

Genotype	WC	Insulin	HOMA	Glucose	Blood Pressure (Systole)	Blood Pressure (Diastole)
**rs4986791(C>T)**						
**CC**	86.10±13.56	11.71±5.70	2.36±1.33	4.98±0.48	118.77±6.89	81.05±8.69
**CT**	85.00±11.04	14.67±5.37	3.86±1.24	4.96±0.31	118.20±4.71	80.40±10.64
***P*-value**	0.357	0.030	0.034	0.190	0.475	0.269
**rs4986790 (A>G)**						
**AA**	85.93±13.08	11.92±5.70	2.99±1.34	4.98±0.48	118.78±6.71	81.78±8.88
**AG+GG**	87.42±13.66	12.80±6.11	3.46±1.37	4.89±0.34	116.33±5.51	74.00±8.54
***P*-value**	0.803	0.254	0.228	0.322	0.552	0.165

Data for variables are shown as mean value with ± SD. Genotypic distribution is found for both co-dominant models using unpaired t-test. Two-tailed *P*-value is significant at ≤ 0.05.

Table S5. Mean values of lipid profile (TC, TG, HDL, LDL) among the study subjects based on the genotype.

GENOTYPE	TC	TG	HDL	LDL
**rs4986791 (C>T)**				
**CC**	4.10±1.16	0.82 ±0.46	1.31±0.42	2.33±1.07
**CT**	3.67±1.35	0.84 ±0.62	1.19±0.35	2.10±1.08
***P*-value**	0.257	0.892	0.246	0.385
**rs4986790 (A>G)**				
**AA**	4.02±1.23	0.81 ±0.41	1.31±0.42	2.34±1.11
**AG + GG**	3.67±0.95	0.91 ±0.79	1.18±0.37	2.06±0.75
***P*-value**	0.197	0.414	0.202	0.247

Data are presented as mean value with ± SD. Genotypic distribution is found for both co-dominant models using unpaired t-test. Two-tailed *P*-value is significant at ≤ 0.05.

Table S6. Estimated haplotype frequency of the co-segregated rs4986791 C>T, and rs4986790 A>G related odds ratios associated with WC as obesity phenotype

Markers	Haplotype	EM Obese Frequency	EM Non-obese Frequency	Odds Ratio	95% Cl	*P*-value
**rs4986791 CT, rs4986790 AG**	CA	0.896	0.891	1.23	0.63- 2.44	0.533
**rs4986791 CT, rs4986790 AG**	CG	0.054	0.053	0.84	0.35- 2.04	0.706
**rs4986791 CT, rs4986790 AG**	TG	0.044	0.038	0.78	0.29- 2.10	0.626

Data are presented as the frequency for non-obese (controls) Vs. Obese subjects (cases) for each haplotype with the odds ratio, lower and upper 95% confidence intervals, and *P*-value. The data are analyzed by logistic regression analysis. ORs are corrected for the Bonferroni test on SNP numbers and FDR. Two-tailed *P*-value is significant <0.05.

<u>Table S7.</u> Estimated haplotype frequency of the co-segregated rs4986791 C>T, and rs4986790 A>G related **odds ratios** associated with HOMA-IR as MS component

Markers	Haplotype	EM Obese Frequency	EM Non-obese Frequency	Odds Ratio	95% Cl	*P*-value
**rs4986791 CT, rs4986790 AG**	CA	0.900	0.862	0.49	0.20- 1.19	0.111
**rs4986791 CT, rs4986790 AG**	CG	0.052	0.051	0.94	0.28- 3.22	0.927
**rs4986791 CT, rs4986790 AG**	TG	0.087	0.019	4.73	1.19- 18.90	0.016

Data are presented as the frequency for non-obese (controls) Vs. Obese subjects (cases) for each haplotype with the odds ratio, lower and upper 95% confidence intervals, and *P*-value. The data are analyzed by logistic regression analysis. ORs are corrected for the Bonferroni test on SNP numbers and FDR. Two-tailed *P*-value is significant <0.05.

Table S8. Estimated haplotype frequency of the co-segregated rs4986791 C>T, and rs4986790 A>G related **odds ratios** associated with **Dyslipidemia**

Markers	Haplotype	EM Obese Frequency	EM Non-obese Frequency	Odds Ratio	95% Cl	*P*-value
**rs4986791 CT, rs4986790 AG**	CA	0.882	0.893	0.83	0.37- 1.86	0.825
**rs4986791 CT, rs4986790 AG**	CG	0.061	0.053	1.14	0.40- 3.27	0.818
**rs4986791 CT, rs4986790 AG**	TG	0.052	0.042	1.23	0.38- 3.93	0.704

Data are presented as the frequency for non-obese (controls) Vs. Obese subjects (cases) for each haplotype with the odds ratio, lower and upper 95% confidence intervals, and *P*-value. The data are analyzed by logistic regression analysis. ORs are corrected for the Bonferroni test on SNP numbers and FDR. Two-tailed *P*-value is significant <0.05.

Table S9. Estimated haplotype frequency of the co-segregated rs4986791 C>T, and rs4986790 A>G related **odds ratios** associated with BMI as obesity phenotype.

Markers	Haplotype	EM Obese Frequency	EM Non-obese Frequency	Odds Ratio	95% Cl	*P*-value
**rs4986791 CT, rs4986790 AG**	CA	0.906	0.888	0.27	0.54- 2.28	0.773
**rs4986791 CT, rs4986790 AG**	CG	0.049	0.058	0.98	0.38- 2.50	0.979
**rs4986791 CT, rs4986790 AG**	TG	0.039	0.048	0.81	0.27- 2.35	0.696

Data are presented as the frequency for non-obese (controls) Vs. Obese subjects (cases) for each haplotype with the odds ratio, lower and upper 95% confidence intervals, and *P*-value. The data are analyzed by logistic regression analysis. ORs are corrected for the Bonferroni test on SNP numbers and FDR. Two-tailed *P*-value is significant <0.05.

Table S10. Estimated haplotype frequency of the co-segregated rs4986791 C>T, and rs4986790 A>G related **odds ratios** associated with hyperglycemia.

Markers	Haplotype	EM Obese Frequency	EM Non-obese Frequency	Odds Ratio	95% Cl	*P*-value
**rs4986791 CT, rs4986790 AG**	CA	0.907	0.871	1.17	0.54-2.24	0.763
**rs4986791 CT, rs4986790 AG**	CG	0.054	0.052	0.99	0.35-2.69	0.958
**rs4986791 CT, rs4986790 AG**	TG	0.038	0.046	0.81	0.22-2.51	0.684

Data are presented as the frequency for non-obese (controls) Vs. Obese subjects (cases) for each haplotype with the odds ratio, lower and upper 95% confidence intervals, and *P*-value. The data are analyzed by logistic regression analysis. ORs are corrected for the Bonferroni test on SNP numbers and FDR. Two-tailed *P*-value is significant <0.05.

S11. (*supplementary table S11)*

Genotype distribution of TLR4^{D299G} and TLR4^{T399I} in different populations

As indicated in Table S5, both TLR4^{D299G} and TLR4^{T399I} are rare mutations in the population of Mixed American (AMR), African American (AFR), and South Asian (EAS).

Table S7. Genotype distribution of TLR4^{D299G} and TLR4^{T399I} in AMR, AFR, and EAS.

Population	Genotype distribution of TLR4^{D299G} (A>G)		Genotype distribution of TLR4^{T399I} (C>T)	
AMR (Mixed American)	A (96.2%)	G (3.8%)	C (96.4%)	T (3.6%)
AFR (African American)	A (92.9%)	G (7.2%)	C (99.4%)	T (0.53%)
EAS (South Asian)	A (87.4%)	G (12.5%)	C (88.3%)	T (11.7%)

Data are presented as percentage (%) of the genotype distribution of both TLR4^{D299G} and TLR4^{T399I}. Data are collected from the National Center for Biotechnology Information (NCBI).

……………………………………………………………………………………..

Table S12. Monocyte cell surface markers used in the study.

Monoclonal antibody	Clone	Fluorochrome	Manufacturer
**HLA-DR**	L243	APC Cy7	BD Biosciences
**CD14**	M5E2	PE	BD Biosciences
**TLR-4 (CD284)**	TF901	PE	BD-Biosciences
**PE MOUSE IgG2K ISOTYPE CONTROL**	MOPC-21	PE	BD-Biosciences

HLA-DR, human leukocyte antigen class II-DR locus; CD, cluster of differentiation; TLR, toll-like receptor; APC Cy7, allophycocyanin combined with cyanine dye; PE Mouse IgG1, Isotype control, PE, R-phycoerythrin. Clone refers to the specified markers of monoclonal antibodies.

Supplementary figures

Fig S1 and S2:

The gating process of flow cytometry for CD284

Monocytes TLR4 were stained with the indicated fluorescent antibodies (PE Mouse Anti-Human TLR4 (CD284) and analyzed in a cell sorter (FACSvantage; BD Biosciences, San Jose, CA, USA). The gate for the monocyte and macrophages population was done according to the cells population size and granularity, cells presented in square gate P5, in Figure S1(A). Unstained cells population was gated in square gate P2, cells presented in Figure S1 (B-D).

Monocytes were stained with the indicated fluorescent antibodies (PE Mouse Anti-Human TLR4 (CD284)) and analyzed in a cell sorter (FACSvantage; BD Biosciences, San Jose, CA, USA). The gate for the monocyte population was done according to the cells population size and granularity, cells presented in square gate P5, in Figure S2 (A). stained cells population was gated in square gate P1 ($x > 10^5$), cells presented in Figure S2 (B-D).

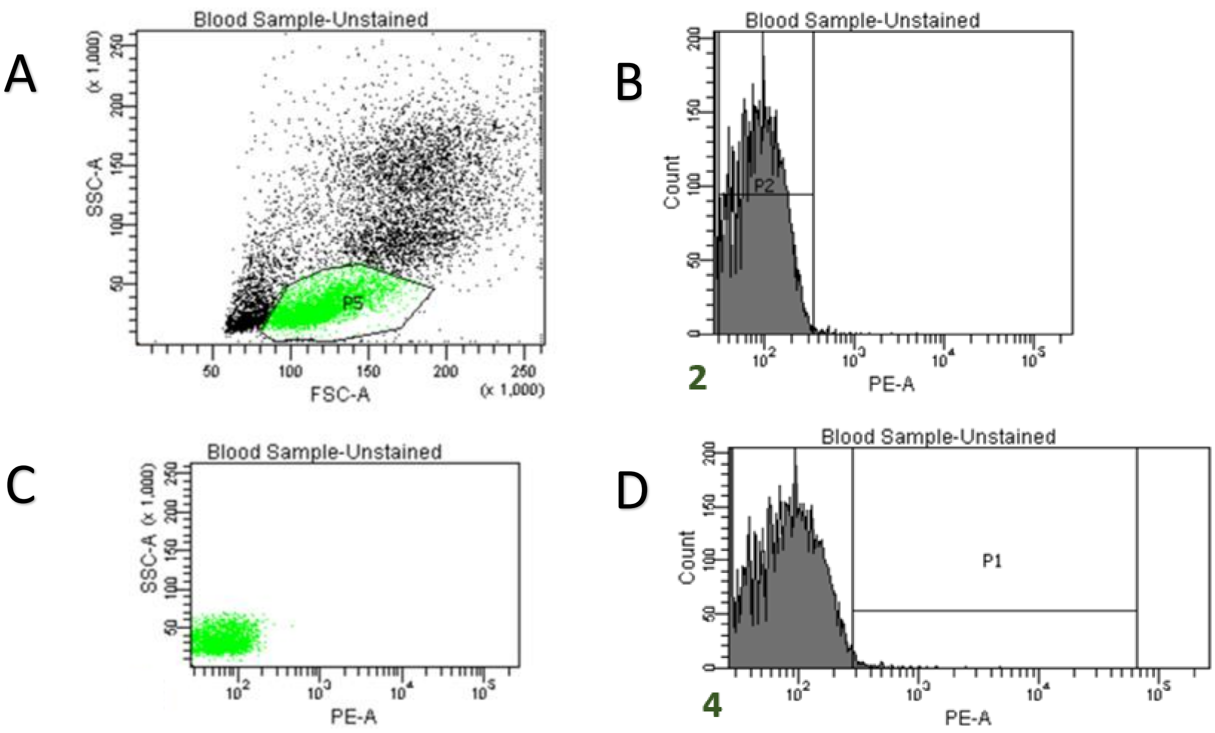

Figure 1. The gating steps for expression of CD284 on Monocytes (unstained cells)

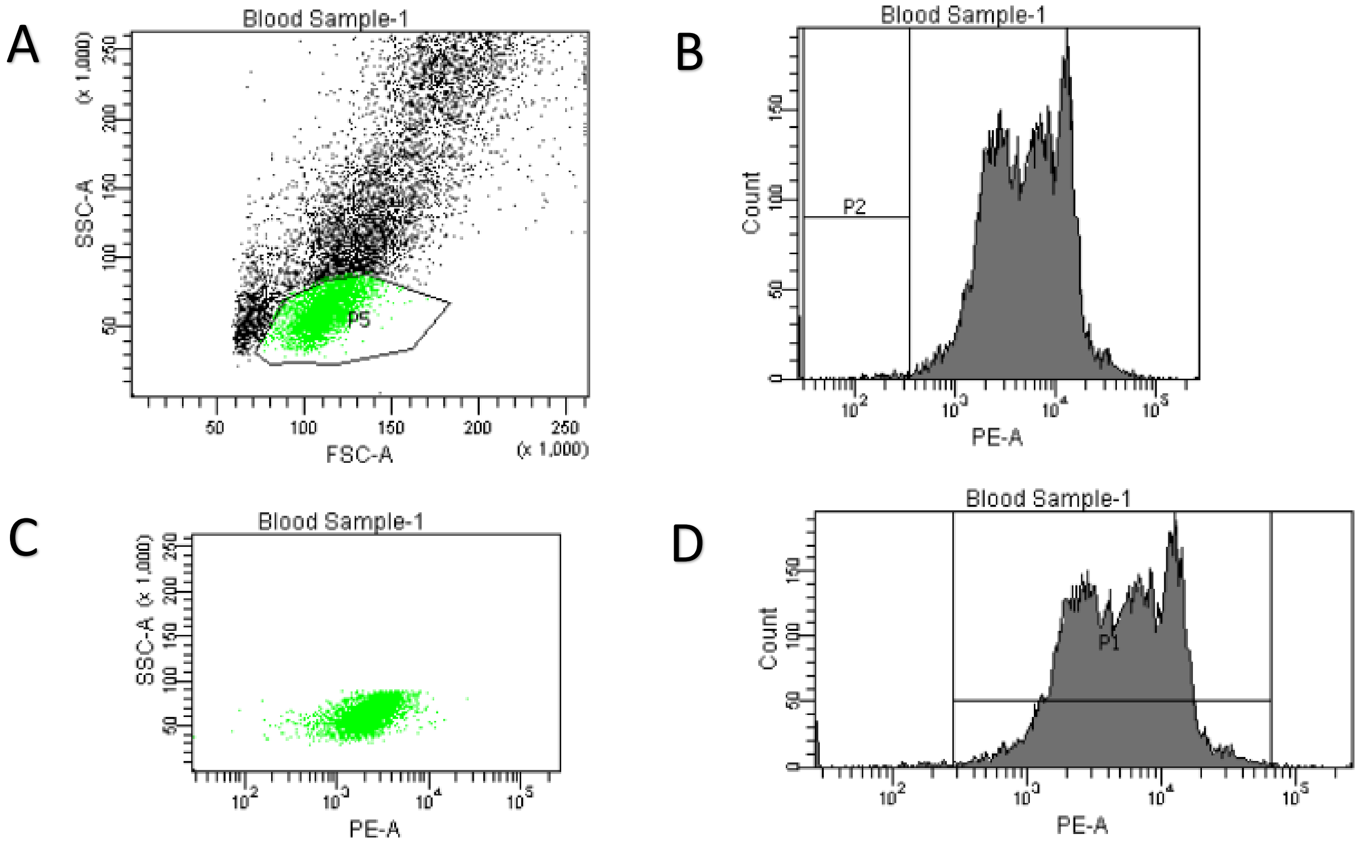

<u>Figure 2.</u> The gating steps for expression of CD284 on Monocytes (stained cells).
